# Immune Interactions Unique to Intradermal Microtoxin: Insights From Aesthetic, Therapeutic, and Vaccination Studies

**DOI:** 10.1111/jocd.71164

**Published:** 2026-09-22

**Authors:** Je‐Young Park, Luis Alberto Parra Hernandez, Hossam Elenany, Martina Kerscher, William Gregory Chernoff, Warner Carr, Samantha Tran

**Affiliations:** ^1^ Apgujeong Oracle Dermatology Clinic Seoul Korea; ^2^ ENCEFALO Latam Scientific Research Group Baranquilla Colombia; ^3^ Sociedad Internacional de Rejuvenecimiento Facial SIRF Baranquilla Colombia; ^4^ PIUBEL Baranquilla Colombia; ^5^ Faculty of Medicine Cairo University Cairo Egypt; ^6^ Division of Cosmetic Sciences University of Hamburg Hamburg Germany; ^7^ Chernoff Plastic Surgery and Laser Centers: Indianapolis (Private Practice) Santa Rosa California USA; ^8^ Department of Surgery Ascension St. Vincent Hospital Indianapolis Indiana USA; ^9^ Allergy & Asthma Associates of Southern California Laguna Niguel California USA; ^10^ Southern California Research Laguna Nigel California USA; ^11^ Merz Aesthetics Raleigh North Carolina USA

**Keywords:** botulinum toxin type A, immunogenicity, intradermal injection, microtoxin, neutralizing antibodies, skin quality

## Abstract

**Introduction:**

Intradermal botulinum toxin type A (BoNT‐A; microtoxin) has been shown to improve skin texture, superficial rhytides, and sweat/sebaceous activity through neuronal (cholinergic) and non‐neuronal pathways. While discussions around immune system interactions for BoNT‐A in aesthetic indications exist for intramuscular injection, the high density of antigen‐presenting cells in the dermis raises the question of whether a different level of concern is warranted.

**Methods:**

This narrative review was informed by a PubMed search through 2025 identifying studies on intradermal BoNT‐A and immunogenicity using combined/related terms for BoNT‐A, intradermal injection, microtoxin, immunogenicity, and skin immunology. Reference lists were screened for additional studies.

**Results:**

The number of publications on microtoxin has increased markedly since 2024. While the skin is an immunologically active organ, immunogenicity data remain limited for aesthetic intradermal injection. To date, only a single prospective study has examined immune responses to intradermal BoNT‐A in aesthetic practice. This study showed higher antibody responses with intradermal versus intramuscular delivery. Like data for intramuscular injection, there is a lack of long‐term data on neutralizing antibody (NAb) development; however, in the context of increasing BoNT‐A doses for facial treatment and increasing long‐term exposure, introduction of BoNT‐A into the dermis over a large surface may raise the risk of NAb development.

**Conclusions:**

Microtoxin techniques may heighten immunogenic potential through dermal antigen exposure and frequent retreatment. IncobotulinumtoxinA is free of complexing proteins and has a low antigenic protein burden, which may favor its use for intradermal injection. However, direct long‐term comparative evidence for aesthetic intradermal treatments is lacking.

## 1 Introduction

Botulinum toxin type A (BoNT‐A) is a mainstay of aesthetic practice, with an established record of safety and efficacy when injected intramuscularly [1, 2, 3]. Increasingly, intradermal ‘microtoxin’ techniques have been adopted for aesthetic purposes, targeting sweat and sebaceous glands, fine dermal musculature, and superficial rhytides, as well as improving horizontal neck lines, enhancing contour, and treating scars and keloids [4, 5]. By reducing repetitive muscle contraction, BoNT‐A may also support skin longevity by decreasing chronic mechanical stress on dermal collagen and elastin, thereby helping limit wrinkle formation [6].

Importantly, the skin is an immunologically active organ enriched with antigen‐presenting cells (APCs). Thus, while microtoxin offers novel therapeutic and aesthetic applications, it also raises important immunological considerations that have not been fully explored in the aesthetic literature. We know from vaccine studies across a range of pathogens that intradermal injection elicits stronger immune responses than intramuscular administration, even at fractional doses, raising the question of whether there is a potential for heightened immune recognition when BoNT‐A is delivered intradermally rather than into muscle [7, 8, 9].

Immunogenicity is a recognized concern in BoNT‐A therapy, as the development of neutralizing antibodies (NAbs) can lead to secondary non‐response (SNR). While this limits aesthetic BoNT‐A efficacy, the greater concern is that it may preclude patients from receiving BoNT‐A as a medical therapy. Meta‐analyses consistently show that NAb formation is highest in therapeutic populations receiving higher cumulative doses and repeat treatment [10]. While rates have historically been considered low for typical “aesthetic doses,” the interest of younger demographics in BoNT‐A treatment, repeated exposure, and emerging use of intradermal techniques warrant careful consideration as lifetime BoNT‐A exposure increases [1]. Importantly, as BoNT‐A use has expanded to include the upper face, lower face, masseters, lower face, and body, doses may be higher than appreciated and can indeed approach that for medical indications where the rate of NAb formation is recognized as 1%–5%. There are currently six BoNT‐A formulations available in the US. While each is effective, it is important to discuss the relative risk of NAb formation. In nature, BoNT‐A associates with accessory proteins such as hemagglutinins, which are not required for clinical efficacy but may act as adjuvants and amplify immune responses [11, 12, 13]. Formulations free of accessory proteins may therefore represent a lower‐risk option for intradermal delivery. This review will examine the immunology of the skin, the implications of intradermal BoNT‐A injection, and whether toxin purity has a role in minimizing treatment resistance.

## 2 Methods

To comprehensively examine intradermal BoNT‐A and its immunogenic implications, we performed a structured literature search in PubMed through 2025, with the objective of providing a narrative synthesis of the evidence. The search was organized across three evidence domains: (1) direct clinical evidence evaluating immune responses or immunogenicity following aesthetic intradermal BoNT‐A administration; (2) supporting mechanistic evidence regarding immune function in the skin, intradermal antigen delivery, and immune responses to intradermal vaccination; and (3) broader clinical evidence regarding BoNT‐A immunogenicity, neutralizing antibody development, SNR, cumulative exposure, and formulation‐related factors.

Specifically, to review aesthetic use of intradermal microtoxin, (“botulinum toxin” OR “BoNT‐A”) AND (“intradermal” OR “microtoxin” OR “microinjection” OR “microbotox” OR “mesobotox”) AND (“aesthetic” OR “cosmetic”) was searched. The search was then narrowed to seek out specific research exploring the immune response to intradermal BoNT‐A injection and included (“botulinum toxin” OR “BoNT‐A”) AND (“intradermal” OR “microtoxin” OR “microinjection” OR “microbotox” OR “mesobotox”) AND (“immunogenicity” OR “neutralizing antibodies” OR “antigen presenting cells” OR “dendritic cells” OR “protein load”), with and without AND (“aesthetic” OR “cosmetic”).

Given the limited direct evidence for aesthetic intradermal BoNT‐A administration, the search was expanded to evaluate general skin immunology, intradermal vaccination, and BoNT‐specific immunization using: ((“skin immunology” OR “cutaneous immune system” OR “antigen presenting cells” OR “dendritic cells” OR “memory T cells”) AND (“intradermal vaccination” OR “cutaneous vaccination” OR “fractional dose”)) OR (“botulinum toxoid vaccine” OR “botulinum toxin antibodies”). Reference lists of included articles were manually screened to capture additional relevant studies.

Publications were considered for inclusion if they contributed relevant clinical or mechanistic evidence regarding intradermal BoNT‐A exposure, cutaneous antigen presentation, intradermal vaccination, BoNT‐specific immune responses, neutralizing antibody development, SNR, cumulative treatment exposure, or formulation‐related immunogenicity. Priority was given to direct clinical studies, systematic reviews and meta‐analyses, and long‐term clinical studies.

## 3 Results

Initial searches to review intradermal microtoxin as an aesthetic procedure revealed studies through 2004. Interestingly, over 25% of microtoxin manuscripts have been published since 2024, reflecting the recent rise in popularity of this technique and underscoring the crucial need to consider potential longer‐term immunological impact. The structured literature search identified a limited body of evidence directly evaluating immunogenicity following aesthetic microtoxin, with only a single study found that specifically examined this, highlighting the need for more research in this area [14]. Removing the search terms ‘AND (“aesthetic” OR “cosmetic”)’ led to inclusion of 2 studies assessing intradermal BoNT‐A injection treatment of axillary hyperhidrosis [15, 16]. Expanding the search beyond microtoxin to general skin immunology, intradermal vaccination, and BoNT‐specific immunization returned 537 unique publications, revealing extensive evidence that the skin is an immunologically active organ and that intradermal antigen delivery consistently produces stronger immune responses than intramuscular injection, even at fractional doses, as a result of the high density of APCs in the dermis. Following screening and review of the literature, 51 references were included in the final narrative review, discussed in the sections below. Table S1 summarizes the key publications used for evidence synthesis.

### 3.1 Microtoxin in Aesthetic Practice

**FIGURE 1:**
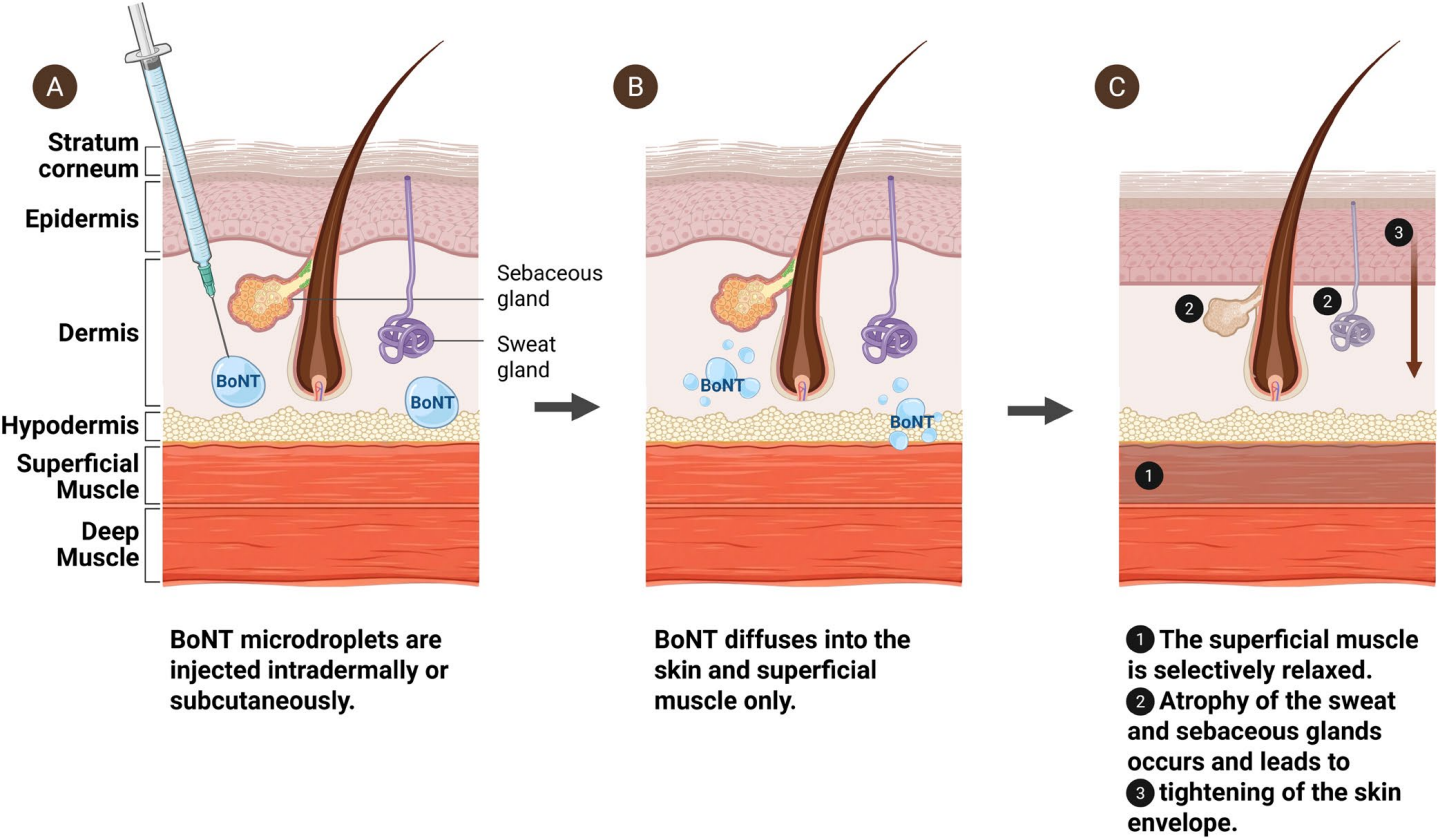
Mechanism of intradermal microtoxin. (A) Microtoxin is delivered as multiple tiny deposits intradermally or at the dermal‐superficial muscle junction. (B) The toxin diffuses locally to the skin and superficial muscle only. (C) Partial denervation of the superficial muscle fibers that insert into the dermis produces subtle relaxation without affecting deeper facial expression. Blocked cholinergic stimulation of the sweat and sebaceous glands reduces perspiration and sebum production. Over time, glandular atrophy and contraction of the skin envelope contribute to improved surface texture and tightening [17] [Created in BioRender. Tran, S. (2026) https://BioRender.com/oajjucp].

Microtoxin techniques have gained rapid adoption in aesthetic medicine. Pioneered by Dr. Woffles Wu in the late 2000s, the microtoxin approach adapted the earlier intramuscular aesthetic applications of botulinum toxin introduced by Drs. Jean and Alastair Carruthers, using highly diluted intradermal injections of BoNT‐A to refine skin texture, reduce pore size and sebum production, and achieve a natural, non‐paralyzing rejuvenation effect (Figure 1) [17, 18, 19]. The technique involves delivering multiple tiny boluses of diluted toxin into the dermis, selectively weakening the superficial fibers of facial muscles that insert into the dermis and contribute to fine dynamic lines, while limiting diffusion to preserve deep muscle activity and natural facial animation [5, 17]. Additional benefits include reduced sweat and sebaceous gland activity, smaller‐appearing pores, improved surface sheen, and overall refined texture [17].

BoNT‐A exerts its aesthetic effects primarily by inhibiting acetylcholine (ACh) release at the neuromuscular junction, causing temporary denervation and relaxation of targeted muscles [20]. Within cutaneous tissues, ACh regulates keratinocyte proliferation, sebogenesis, pigmentation, microcirculation, and inflammatory responses, providing the biologic basis for BoNT‐A use in rosacea, acne, seborrhea, and general skin rejuvenation [4, 21]. Blockade of cholinergic stimulation of sweat and sebaceous glands reduces perspiration and sebum production, enhancing texture, clarity, and luster [5, 22]. Additional effects include modulation of fibroblast activity and collagen remodeling, improving wound healing and reducing hypertrophic scarring [4, 23]. BoNT‐A may also enhance dermal hydration through relaxation of perilymphatic musculature and transient osmotic shifts, contributing to the luminous and tightened appearance often observed for approximately 3 months [5]. In addition, selective weakening of superficial facial depressor muscles, including the platysma, depressor anguli oris, and orbital portion of the orbicularis oculi, reduces downward vector forces on the lower face and periorbital region, permitting unopposed elevator muscle activity and contributing to subtle jawline sharpening, oral commissure elevation, and brow lifting [24]. Collectively, neuromodulatory, glandular, and microvascular mechanisms underpin the expanding use of microtoxin for superficial rhytids, enlarged pores, seborrhea, rosacea, and subtle lifting or contour refinement [5].

The microtoxin technique is generally well tolerated and associated with a favorable safety profile [25]. Compared with traditional intramuscular injections, the lower toxin concentrations and shallower injection depth reduce the risk of diffusion to unintended muscles (Table 1) [26, 27]. This results in a lower incidence of complications in challenging regions such as the lower forehead and infraorbital areas, where deep injection risks brow or lid ptosis, ectropion, or asymmetric smile [25]. Nevertheless, complications can occur when microdroplets are too large or inadvertently delivered into the subdermis, leading to unintended diffusion into underlying muscle and temporary paralysis [17, 27]. Superficial wheal formation during intradermal injection can also cause transient discomfort due to multiple needle punctures and dermal fluid distension [26]. Patients typically report greater injection‐site discomfort than with standard BoNT‐A, although this can be mitigated with topical or mixed lidocaine anesthesia and adjunctive cooling measures, including cold air application [28]. Importantly, the superficial approach results in minimal bruising and a reduced incidence of deep‐muscle adverse effects compared with traditional injections [25].

**TABLE 1:** Key differences between conventional BoNT‐A and intradermal microtoxin techniques [4, 17, 19].

Feature	Conventional BoNT‐A (intramuscular BoNT‐A)	Microtoxin (intradermal BoNT‐A)
Primary indications	Dynamic wrinkles via skeletal muscle relaxation	Superficial rhytides via modulation of dermal musculature, skin texture improvement, pore and sebum reduction, rosacea, acne, and prevention or treatment of hypertrophic scars
Injection plane	Intramuscular	Intradermal or dermal–subdermal interface
Dilution	~100 U per 2.5 mL (standard)	Highly diluted (20–30 U per mL)
Duration of Effect	~3–6 months	~2–3 months
Immunologic considerations	Limited dermal antigen exposure; minimal short‐term immune activation	Repeated exposure in an immunologically active tissue may enhance antibody formation risk

Although the aesthetic benefits of intradermal BoNT‐A are well established, its immunologic implications remain insufficiently studied. The high frequency of repeat treatments, cumulative dosing, and the immunologically active nature of the dermis raise concern for antibody formation, emphasizing the importance of formulation purity, injection interval, and continued study of immunogenic outcomes.

### 3.2 Dermal Immune Mechanisms: Evidence From Vaccination Models

#### 3.2.1 The Skin Is an Immunologically Enriched Organ

**FIGURE 2:**
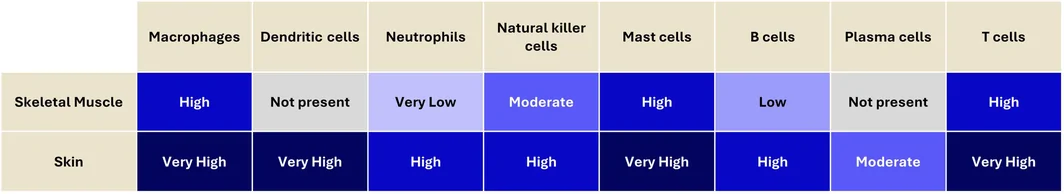
Immune cell densities in healthy human skin compared to skeletal muscle. Based on data from Sender et al. [29]. Immune cell densities (cells per gram of tissue) for each cell type were obtained from the density values reported by Sender et al. [27]. Values were log_10_‐transformed and categorized based on the classification: Very High (≥ 10^6^), High (10^5^), Moderate (10^4^), Low (10^3^), and Very Low (10^2^). Dendritic cells and plasma cells were designated “Not present” in skeletal muscle because (i) these cell types were not quantified by Sender et al. and (ii) established histology sources indicate that they are not typical residents of healthy skeletal muscle, appearing primarily in pathological infiltrates such as inflammatory myopathies.

The skin is an immunologically active barrier epithelial organ. As the body's first line of defense against pathogens, skin plays a central role in both innate and adaptive immune defense. Quantitative immune‐cell mapping confirms that the skin contains high densities of dendritic cells, T cells, mast cells, and macrophages, present at levels several orders of magnitude greater than that found in muscle, underscoring the heightened immunologic activity of the skin (Figure 2) [29]. It contains an extensive network of resident and migratory immune cells that continually survey for foreign antigens and environmental insults. Among these, Langerhans cells in the epidermis and dendritic cells in the dermis are potent APCs capable of capturing, processing, and presenting antigens to draining lymph nodes, thereby initiating both humoral and cellular immune responses [7, 30]. Beyond APCs, the skin houses specialized barrier‐defense lymphocytes, γδ T cells, that are enriched in epidermis and dermis. These cells rapidly produce cytokines (e.g., IL‐17) and growth factors that modulate inflammation, wound repair, and antimicrobial defense, underscoring the dermis's heightened immune reactivity [24]. Intradermal injection introduces antigens directly into this APC‐rich environment, enabling rapid antigen capture and migration of dendritic cells to regional lymph nodes where adaptive immune priming occurs [7, 30].

#### 3.2.2 Dermal Response to Vaccination

Consistent with the heightened number of APCs in the skin, numerous vaccination studies have demonstrated that intradermal administration can elicit immune responses superior to those induced by intramuscular injection, even when fractional antigen doses are used [7, 8, 9, 25]. This finding is consistent across vaccination systems and antigens: a systematic review and meta‐analysis of 45 studies found that intradermal dosing at ~20%–60% of standard antigen was non‐inferior for influenza, rabies, and hepatitis B vaccines [25]. The authors concluded that intradermal delivery enables dose‐sparing without loss of immunogenicity for several vaccines [7, 25]. Similarly, a comprehensive review of intradermal versus intramuscular vaccination concluded that intradermal immunization generally generates greater immune responses than intramuscular injection [7]. The authors attributed these observations to the dermis's rich network of dendritic cells. Antigen capture in skin, coupled with local inflammation, promotes dendritic‐cell maturation and trafficking to draining lymph nodes, improving priming efficiency [7]. Clinically, modification of vaccination administration to include intradermal delivery been used successfully to overcome suboptimal intramuscular responses in special patient populations, such as patients receiving hepatitis B vaccination during dialysis [26]. In addition, intradermal vaccination produces a more durable T‐cell response, including more robust CD8^+^ effector and memory T‐cell generation than standard intramuscular dosing [27]. Thus, intradermal delivery can efficiently prime both arms of adaptive immunity against antigens.

#### 3.2.3 Dermal Response to BoNT Antigens

Preclinical investigations have demonstrated that the skin's high immunogenic potential extends to BoNT. In a murine model, intradermal electroporation of a DNA vaccine encoding the C‐terminal heavy‐chain fragment of BoNT serotype E induced both robust humoral and cellular immunity [30]. Repeated intradermal doses generated high BoNT‐specific IgG titers and strong interferon‐γ–secreting T‐cell responses, which together conferred complete protection against lethal BoNT/E challenge [30]. Similarly, in non‐human primates, intradermal microneedle administration of recombinant BoNT/A binding fragments elicited potent neutralizing antibody responses and fully protected vaccinated macaques from otherwise lethal aerosolized toxin exposure [28]. Although these studies demonstrate the skin's capacity to mount robust immune responses to BoNT antigens, they employed vaccine constructs specifically designed to maximize immune activation. However, these findings do confirm that intradermal delivery can effectively prime both arms of adaptive immunity against BoNT antigens, highlighting the dermis as a highly efficient platform for systemic immunization even at fractional antigen doses. These data underscore that the dermal immune network is capable of recognizing and mounting a response to BoNT antigens themselves, raising the practical concern that repeated intradermal microtoxin exposure could engage similar immune mechanisms over time.

### 3.3 Immunogenicity of BoNT‐A

A key safety concern for any protein‐based therapy is the development of neutralizing antibodies. For BoNT‐A, the toxin itself, as well as associated non‐toxin proteins, have the ability to elicit an immune response. For BoNT‐A, the development of NAbs can lead to SNR [31], which may compromise BoNT‐A responsiveness in therapeutic settings such as cervical dystonia and chronic migraine [32, 33, 34]. For example, in cervical dystonia, approximately 20% of patients discontinue BoNT therapy, most commonly after progressive treatment failure, typically emerging within 3 years of ongoing treatment [35, 36]. To date, no studies have addressed whether prior long‐term use of aesthetic BoNT‐A predisposes individuals to later development of non‐response; however, the therapeutic benefit of BoNT is likely to expand to more clinical conditions in the future, highlighting the importance of preserving its activity in patients [34].

**FIGURE 3:**
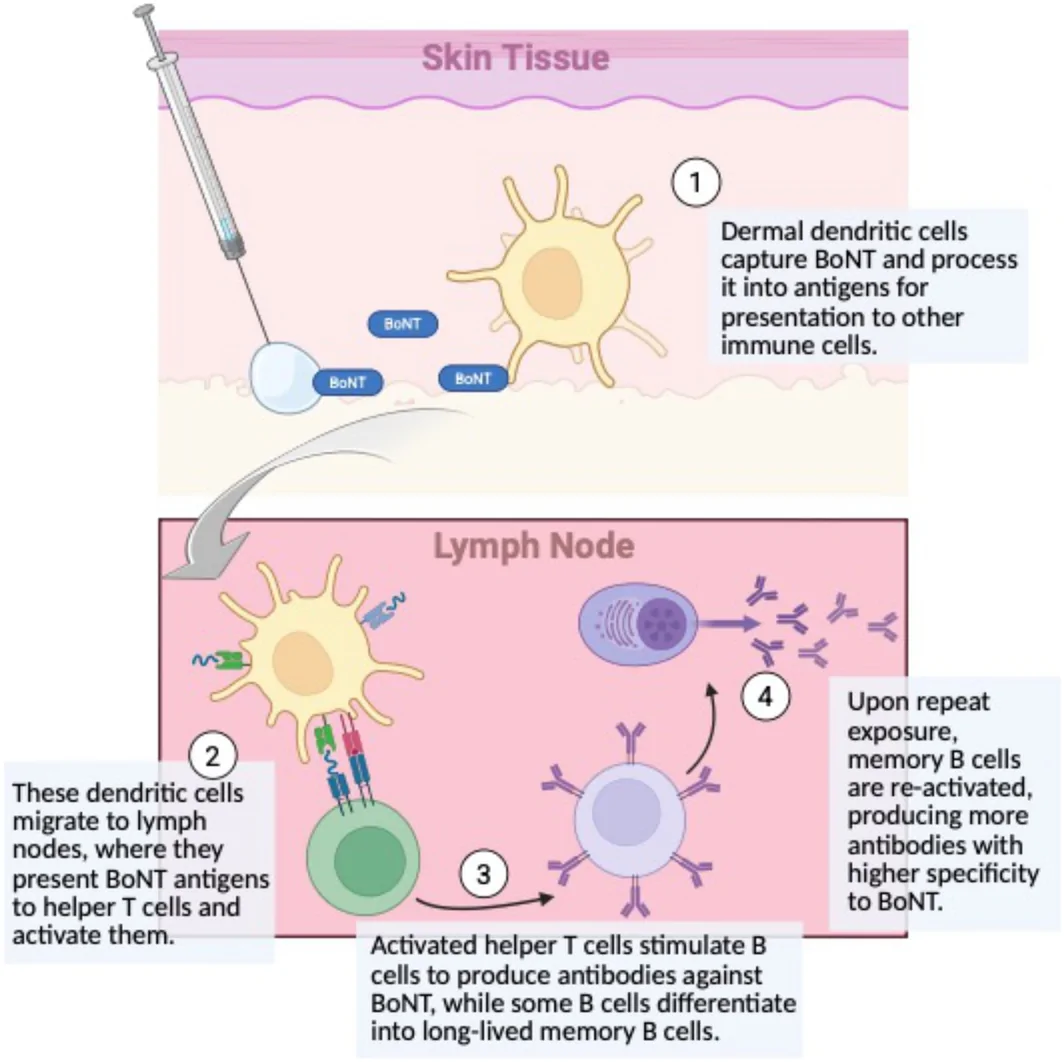
Immunologic cascade leading to neutralizing antibody (NAb) formation against BoNT‐A. (1) Dermal dendritic cells capture BoNT and process it into antigens for presentation to other immune cells. (2) These dendritic cells migrate to lymph nodes, where they present BoNT antigens to helper T cells and activate them. (3) Activated helper T cells stimulate B cells to produce antibodies against BoNT, while some B cells differentiate into long‐lived memory B cells. (4) Upon repeated exposure, memory B cells are re‐activated, producing more antibodies with higher specificity to BoNT [Created in BioRender. Tran, S. (2026) https://BioRender.com/6jdj1b3].

Neutralizing antibody formation is initiated when dendritic cells recognize foreign antigens as “danger signals” through Toll‐like receptors, phagocytose them, and migrate to lymph nodes to activate naïve T‐helper cells (Figure 3). Activated T cells, in turn, stimulate antigen‐specific B‐cell expansion and antibody production against BoNT‐A [32]. While BoNT‐A is capable of activating dendritic cells, non‐toxin proteins present in different formulations, including hemagglutinins (HAs) and non‐HAs, may amplify this response [11, 13]. Complexing proteins, which are produced along with the native toxin and remain bound to the toxin unless removed during the purification process, along with residual impurities such as flagellin, denatured toxin, or clostridial DNA, can function as adjuvants, triggering Toll‐like receptor activation and amplifying immune responses [37]. In one preclinical study on botulinum toxin type B, the presence of hemagglutinin proteins HA1 and HA3 resulted in a greater production of neutralizing antibodies against BoNT‐B, indicating an adjuvant activity for these accessory proteins [38].

Clinically, SNR may manifest as either partial or complete loss of efficacy. In partial SNR, patients experience “dose creep,” requiring higher doses for equivalent results, or “interval creep,” in which effect duration shortens over time [39]. Complete SNR reflects total treatment failure despite adequate dosing. Subtle manifestations such as dose or interval creep are often overlooked in aesthetic practice, leading to underrecognition of BoNT‐A immunogenicity [40]. Recognition is further complicated by incomplete treatment records, as aesthetic patients frequently receive BoNT‐A from multiple providers [40].

While short‐term studies often report a relatively low NAb incidence, these data reflect limited exposure windows, generally with less than 2 years [41]. However, longer‐term data suggest that cumulative prevalence across medical indications may exceed 15%–20% after 10–20 years of continuous BoNT/A therapy [39, 40]. The lack of longer term studies is important: in one meta‐analysis of 43 studies (*n* = 8833) across therapeutic indications, treatment duration emerging as a significant predictor of antibody development (*p* = 0.007) and a higher NAb prevalence of 1.8% (95% CI, 1.2%–2.3%) was reported [42]. Notably, meta‐regression identified duration > 10 years as a stronger correlate of NAb formation than mean dose or injection frequency, suggesting that cumulative exposure over time, rather than individual session intensity, drives immunogenicity. Rates of NAbs were greatest among patients with dystonia (7.4%), spasticity (6.7%), and urologic indications (6.2%). Across formulations, abobotulinumtoxinA (AboA) showed the highest NAb rate (7.4%), whereas incobotulinumtoxinA (IncoA) and onabotulinumtoxinA (OnaA) exhibited markedly lower rates (~0.3%) [42]. Similarly, in a cohort of 645 long‐term BoNT/A–treated patients, Hefter et al. reported no NAbs among those treated exclusively with IncoA, compared with 5.9% among patients maintained on complex‐protein formulations and 33.3% among those who switched products (*p* < 0.035) [39]. In a cervical dystonia cohort of 130 patients, the original high‐protein OnaA (25 ng/100 U) produced NAbs in 9.5% of patients, whereas no antibodies were detected in those treated exclusively with the current low‐protein preparation (5 ng/100 U), representing a six‐fold reduction in risk [43]. These findings were observed over the course of several months, but nevertheless support the importance of minimizing impurities.

These findings are consistent with the known molecular differences among formulations, as highly purified formulations containing only the 150‐kDa core neurotoxin, such as IncoA, lack NAPs and are less likely to elicit neutralizing antibodies [32, 37]. Clinical evidence also supports this reduced immunogenicity. In high‐dose spasticity studies, IncoA demonstrated no cases of SNR even at cumulative doses up to 800 U [44]. Furthermore, in aesthetic cohorts, switching to IncoA after SNR has been advocated by experts and has been associated with declining NAb titers and clinical response, further underscoring its comparatively low immunogenic profile [45].

Immunogenic risk is also shaped by the overall treatment pattern, including dose, cumulative exposure, and retreatment intervals. Short retreatment intervals, booster dosing within 3 weeks, and high cumulative exposure increase the likelihood of immune sensitization [31, 32]. Although aesthetic doses are individually small, repeated injections across multiple treatment areas can approximate therapeutic dosing levels [32, 46].

### 3.4 Clinical Implications for Microtoxin

From an immunologic perspective, the dermis poses unique challenges for BoNT‐A due to its greater density of dendritic cells and heightened capacity for antigen capture [7]. In addition, though microtoxin injections use lower unit doses than conventional intramuscular treatments, their shorter duration of effect (typically 3 months) necessitates more frequent retreatment, increasing lifetime antigen exposure [47, 48].

To minimize immunogenic risk, experts favor use of the purest available formulations for both treatment‐naïve patients and those experiencing diminished response [31]. Given the high frequency and cumulative exposure inherent to intradermal microtoxin use, the selection of a low‐immunogenic formulation is critical for maintaining efficacy and durability across repeated aesthetic treatments. Cumulative exposure and repeated intradermal dosing could meaningfully increase long‐term immunogenic risk, underscoring the importance of formulation purity and adequate retreatment intervals [49]. Initiating treatment with a highly purified, complex‐protein‐free formulation such as IncoA can meaningfully reduce the risk of immune sensitization from the outset, an important consideration for aesthetic protocols that rely on repeated or multi‐site dosing [39]. Once antibodies develop under complex‐protein exposure, the risk is not fully reversible, although switching to IncoA can allow a gradual return to clinical responsiveness [39, 50]. Moreover, higher‐dose regimens of IncoA have been well tolerated in therapeutic settings, with no secondary nonresponse due to neutralizing antibodies reported in the TOWER study at doses up to 800 U [44]. Given the substantial rates of extrapolated antibody prevalence after 10–20 years of continuous BoNT/A therapy, formulation selection and dosing strategy are likely to influence the long‐term durability of aesthetic outcomes.

In a multicenter trial of intradermal OnaA for axillary hyperhidrosis, neutralizing antibodies were largely absent over the 16‐month study period, with only one patient showing seroconversion to antibody‐positive at study end. However, this individual remained clinically responsive and reverted to antibody‐negative status on subsequent testing. Interpretation of these findings is limited by short study duration and restricted treatment exposure, as most participants received only one or two intradermal treatments [15].

Our search identified the only study to date specifically examining immune responses to aesthetic intradermal BoNT‐A injection, published in 2025 [14]. This small pilot study evaluated intradermal versus intramuscular injection of several BoNT‐A formulations with 180‐day IgG follow‐up, though conclusions are limited by small subgroup sizes, short duration, and the absence of validation against a functional neutralization assay such as the mouse hemidiaphragm assay. When all products were analyzed together, intradermal administration produced a stronger humoral response than intramuscular injection, with ELISA testing showing significantly elevated IgG at several time points. Several formulations, such as AboA and prabotulinumtoxinA (PraboA), showed clear intradermal elevation. IncoA was the only product besides letibotulinumtoxinA (LetiA) whose intradermal median never rose above 1.0, and its intramuscular values remained consistently below baseline, indicating the lowest immunogenic signal in the study. Although LetiA appeared unique in showing no intradermal increase, supplemental analyses revealed that this pattern resulted from variability between two manufacturing sources rather than a consistently low‐immunogenicity profile. Intradermal administration produced a statistically significant day‐30 rise for AboA, PraboA, and LetiA, whereas neither IncoA nor OnaA showed significant increases from baseline; however, IncoA exhibited the lowest functional‐site immunogenicity across routes [14]. While limited by single‐injection treatments and short follow‐up, the trend toward higher total IgG with intradermal versus intramuscular injection underscores the need for careful consideration of treatment plans to mitigate immune activation in microtoxin applications.

## 4 Discussion

Immunogenicity remains an underrecognized yet increasingly relevant concern in aesthetic BoNT‐A use. Meta‐analyses such as Jankovic et al. provide valuable incidence data but are constrained by short trial durations and lack of continuous patient follow‐up, potentially capturing only early antibody formation rather than true prevalence [41]. Although participants were followed for up to 15 treatment cycles, nearly 95% completed nine or fewer, equating to about 2–3 years of exposure, and aesthetic cohorts rarely exceeded five cycles (~1.5 years).

Given that antibody formation is a cumulative process that typically manifests after years of repeated dosing, the absence of neutralizing NAbs within limited time frames cannot exclude later seroconversion [39]. Real‐world observational data indicate that treatment duration and cumulative exposure are the dominant drivers of immunogenicity, with substantial NAb prevalence after one to two decades of continuous therapy [39, 40]. In the Rahman et al. meta‐analysis, which incorporated longer‐term therapeutic datasets, treatment duration emerged as a statistically significant predictor of antibody formation, while mean session dose and injection number did not [42]. The authors proposed that cumulative exposure over extended treatment spans, often accompanied by gradual dose escalation to maintain efficacy, contributes more to immune sensitization than any single high‐dose exposure [42]. This pattern aligns with clinical observations that adaptive neuroplasticity in treated muscles can necessitate progressive dose escalation to maintain efficacy, further increasing lifetime antigen exposure and cumulative immunogenic risk [36, 42]. Together, long‐term studies reinforce that cumulative exposure and extended duration, rather than acute dosing intensity, govern BoNT‐A immunogenicity. Because no registries or standardized antibody testing frameworks exist in aesthetic practice, the actual incidence of immunogenicity will likely remain unknown; however, studies with longer follow‐up and sensitive binding antibody assays are encouraged to help elucidate the lifetime immunogenic risk in aesthetic practice.

Within a skin longevity framework, the benefits of BoNT‐A may extend beyond wrinkle reduction, as decreased repetitive muscle movement could lessen cumulative mechanical stress on collagen‐ and elastin‐rich dermal structures [6]. As aesthetic practice evolves toward multi‐area injection and shorter retreatment intervals, total cumulative doses increasingly approximate those used in therapeutic indications, where NAb prevalence exceeds the low rate historically cited for cosmetic use. The studies discussed here confirm that dose intensity, treatment frequency, cumulative exposure, and treatment duration all contribute to neutralizing antibody risk [32, 33, 34, 35, 49]. While per‐session aesthetic doses are often perceived as low, the dose can easily exceed 100 U depending on the treatment area and whether single or multiple indications are addressed in a given session. Wu's microtoxin protocol uses approximately 80–120 U for full‐face and neck treatment, and body‐contouring indications such as trapezius or gastrocnemius injections can exceed 100–700 U [17, 46]. Consequently, repeated multi‐site exposure, particularly with complex protein‐containing formulations, may cumulatively heighten immunogenic potential. Importantly, both clinical and mechanistic evidence demonstrate that patients who develop SNR on complex protein‐containing formulations may regain efficacy after switching to highly purified, protein‐free preparations such as IncoA, supporting a causal role of accessory proteins in immune activation [50, 51].

The implications extend to intradermal microtoxin applications, where injection occurs into an immunologically active tissue compartment rich in APCs. Vaccination studies have consistently shown that intradermal delivery elicits stronger immune responses than intramuscular injection, even at fractional doses, due to efficient antigen capture by dermal dendritic cells and enhanced migration to lymph nodes. Consistent with this finding, Srinoulprasert et al. demonstrated higher antibody titers following intradermal versus intramuscular administration with lower doses (50 U for all tested BoNT‐As, with the exception of AboA where the dose was 150 U), with the strongest immune activation observed in formulations containing complexing proteins. In contrast, IncoA, which lacks accessory proteins, produced the weakest overall antibody response and even showed a decline from baseline following intramuscular injection. Although the clinical impact was limited by small doses and short follow‐up, these findings underscore that while microtoxin injections employ lower per‐session units, their higher frequency and immunogenic injection site may amplify the risk of sensitization over time.

Across studies, both IncoA and OnaA demonstrate consistently low antigenicity, particularly compared with formulations such as AboA or PraboA [14]. Like AboA and PraboA, OnaA is also a complex‐containing formulation but has shown low rates of NAb formation across pooled analyses and multicenter trials [15, 41]. However, long‐term cross‐sectional data suggest that IncoA may offer an additional margin of immunologic safety, with no NAbs detected among patients exclusively treated with this complex protein‐free formulation, even after years of continuous therapy. Standardization of intradermal technique represents another unmet need, as variations in injection depth, dilution ratios, and microdroplet volume can influence both diffusion patterns and antigen exposure, potentially affecting immunologic outcomes.

Finally, there is a critical opportunity to translate immunogenicity insights from therapeutic literature into aesthetic education. Clinicians should be aware that formulation purity, injection interval, and cumulative exposure are interdependent determinants of immune tolerance. Patient counseling can reinforce that while BoNT‐A remains safe and highly effective, thoughtful selection of formulation and dosing strategy is key to preserving long‐term responsiveness and treatment durability, particularly for microtoxin procedures involving the immunologically active dermis.

Interpretation of these findings is limited by heterogeneity in study design and antibody testing methodology, as well as the shortage of long‐term prospective data in aesthetic populations. Direct evidence regarding the immunogenic implications of aesthetic intradermal BoNT‐A administration is limited to a single prospective pilot study, leaving much of the current synthesis informed by evidence from intradermal vaccination studies, preclinical BoNT immunization models, and therapeutic BoNT‐A populations. Although these studies provide relevant biologic and clinical context, their findings cannot be directly extrapolated to aesthetic microtoxin treatment. It is also important to consider that vaccines are specifically designed to elicit immune responses, whereas therapeutic BoNT‐A is administered in a different clinical context using different formulations, doses, and treatment patterns.

The pilot study evaluating intradermal versus intramuscular BoNT‐A administration was limited by its small treatment groups, short follow‐up duration, and evaluation of only a single treatment exposure [14]. Furthermore, the study evaluated total BoNT/A‐reactive IgG and IgG directed against functional domains and complexing proteins. These binding antibody responses indicate immune recognition but do not establish the presence of functionally neutralizing antibodies or predict clinically meaningful treatment resistance [49]. Non‐neutralizing binding antibodies may recognize BoNT‐A without affecting its biologic activity, whereas NAbs can interfere with toxin activity and are therefore more directly relevant to SNR [49]. Thus, while the available evidence suggests that intradermal administration may influence immune recognition, it does not demonstrate an increased risk of NAb formation or clinically meaningful SNR following aesthetic microtoxin treatment. Prospective longitudinal studies incorporating repeated treatment exposure, validated functional NAb assays, such as the mouse hemidiaphragm assay, and clinical response assessments are needed to determine whether the observed immunologic signals translate into clinically meaningful outcomes.

## 5 Conclusion

Intradermal microtoxin techniques represent an evolving extension of aesthetic BoNT‐A practice, offering novel benefits but introducing distinct immunologic considerations. Current evidence indicates that antibody formation is shaped by cumulative exposure, treatment duration, and formulation purity. Among available formulations, IncoA exhibits the lowest antigenicity, with long‐term data supporting IncoA's protein‐free composition as the most favorable immunologic safety profile. However, whether these characteristics translate into a lower risk of clinically meaningful immunogenicity following aesthetic intradermal administration remains unclear. Given the dermis' heightened immune responsiveness and the increasing frequency of aesthetic retreatment, further prospective studies are needed to define long‐term immunogenic risk in microtoxin applications and to establish standardized injection protocols that preserve efficacy over time.

## Author Contributions

W.G.C., H.E., J.‐Y.P., L.A.P.H., M.K., S.T., W.C. contributed to the conception of the study and discussion points. G.V. and B.S., listed in the acknowledgements, drafted the paper, and W.G.C., H.E., J.‐Y.P., L.A.P.H., M.K., S.T., and W.C., each revised and approved the final manuscript.

## Funding

Funding for medical writing support was provided by Merz North America Inc.

## Disclosure

Je‐Young Park: Speaker and consultant for Merz Aesthetics. Luis Alberto Parra Hernandez: Speaker for Merz Aesthetics. Hossam Elenany: Speaker and trainer for Merz Aesthetics. Martina Kerscher: Advisory Board member, speaker, and clinical investigator (with Hamburg University Research Institute) for Allergan/Abbvie, Croma, Ipsen Innovation, Merz Aesthetics. William Gregory Chernoff: Consultant for Allergan/Abbvie, Galderma, Merz Aesthetics, and Revance. Warner Carr: Speaker and consultant for Merz Therapeutics. Samantha Tran: Employee of Merz Aesthetics.

## Conflicts of Interest

The authors declare no conflicts of interest.

## Supporting information


**Table S1:** Key evidence informing the potential immunologic implications of intradermal BoNT‐A.

## Data Availability

Data sharing not applicable to this article as no datasets were generated or analysed during the current study.
