# Supplementary material for: Immune Interactions Unique to Intradermal Microtoxin: Insights From Aesthetic, Therapeutic, and Vaccination Studies
Source: J Cosmet Dermatol. 2026 Sep 22;25(10):e71164. doi: 10.1111/jocd.71164 (PMC13595894; doi:10.1111/jocd.71164)
Supplement: Supplementary file 1 [file JOCD-25-e71164-s001.docx]

**Supplementary Table 1: Key evidence informing the potential immunologic implications of intradermal BoNT-A**

| **Evidence Domain*** | **Reference** | **Study Design** | **Relevance** |
| --- | --- | --- | --- |
| Direct clinical evidence | Srinoulprasert et al., 2025^14^ | Prospective comparative pilot study | Only study directly comparing Ab responses after aesthetic intradermal versus intramuscular BoNT-A |
|  | Naumann et al., 2003^15^ | Prospective multicenter clinical study | Evaluated Ab development following repeated intradermal OnaA treatment for axillary hyperhidrosis |
|  | Wollina et al., 2002^16^ | Prospective clinical study | Evaluated high-dose intradermal BoNT-A treatment for axillary hyperhidrosis |
| Supporting mechanistic evidence | Sender et al., 2023^24^ | Quantitative analysis based on a literature survey | Characterized immune-cell density in skin relative to other tissues |
|  | Schnyder et al., 2020^27^ | Systematic review and meta-analysis | Evaluated the immunogenicity of fractional intradermal versus standard vaccination |
|  | Zhang et al., 2015^7^ | Comprehensive review | Synthesized immunologic effects of intradermal versus intramuscular vaccine administration |
|  | Della Cioppa et al., 2014^8^ | Clinical dose-ranging study | Compared immune responses following intradermal and intramuscular influenza vaccination |
|  | Troy et al., 2015^9^ | Comparative clinical study | Compared immune responses following intradermal and intramuscular polio vaccination |
|  | Barraclough et al., 2009^28^ | Prospective randomized clinical trial | Evaluated intradermal versus intramuscular hepatitis B vaccination in prior nonresponders |
|  | Sophonmanee et al., 2024^29^ | Clinical follow-up study | Evaluated memory T-cell responses following intradermal vaccination |
|  | Kim et al., 2019^25^ | Preclinical murine study | Demonstrated humoral and cellular responses following intradermal delivery of a BoNT-E vaccine |
|  | Lee et al., 2005^38^ | Preclinical murine study | Demonstrated that BoNT-B proteins HA1 and HA3b enhanced NAb production |
|  | Morefield et al., 2008^30^ | Preclinical primate study | Demonstrated neutralizing antibody responses following intradermal delivery of BoNT-derived antigens |
| Broader clinical evidence | Fabbri et al., 2016^10^ | Systematic review and meta-analysis | Evaluated NAb development across therapeutic BoNT populations |
|  | Rahman et al., 2022^42^ | Systematic review and meta-analysis | Evaluated BoNT-A NAb prevalence and associations with indication, treatment duration, and formulation |
|  | Jankovic et al., 2023^39^ | Pooled meta-analysis of clinical trials | Evaluated NAb development following OnaA treatment across multiple indications |
|  | Albrecht et al., 2019^41^ | Long-term cross-sectional study | Evaluated NAb prevalence following prolonged BoNT-A treatment |
|  | Hefter et al., 2023^40^ | Long-term cross-sectional study | Compared NAb prevalence according to BoNT-A treatment history and formulation |
|  | Jankovic et al., 2003^43^ | Comparative clinical study | Evaluated the relationship between OnaA formulations and immunogenicity |
|  | Wissel et al., 2017^44^ | Prospective multicenter dose-escalation study | Evaluated high-dose IncoA treatment, including immunogenicity outcomes |
|  | Wanitphakdeedecha et al., 2020^51^ | Clinical observational study | Evaluated antibody responses in aesthetic patients with secondary treatment failure |
|  | Ho et al., 2022^32^ | Multidisciplinary review and consensus | Synthesized evidence and expert recommendations regarding BoNT-A resistance |
|  | Park et al., 2022^31^ | Physician survey and expert recommendations | Characterized clinical experiences with aesthetic BoNT-A immunogenicity and SNR |
|  | Bellows and Jankovic, 2019^49^ | Narrative review | Summarized risk factors, antibody assessment, and clinical implications of BoNT immunogenicity |
|  | Kroumpouzos and Silikovich, 2025^45^ | Narrative review | Reviewed causes and management of treatment nonresponse in aesthetic practice |

*Direct clinical evidence includes studies evaluating intradermal BoNT-A administration in aesthetic or other clinical settings. Supporting mechanistic evidence includes studies of cutaneous immune-cell distribution, intradermal vaccination, and BoNT-specific intradermal immunization. Broader clinical evidence includes studies evaluating BoNT-A immunogenicity, NAb development, SNR, cumulative exposure, and formulation-related factors.

BoNT, botulinum neurotoxin; BoNT-A, botulinum neurotoxin type A; BoNT-E, botulinum neurotoxin type E; HA, hemagglutinin; IncoA, incobotulinumtoxinA; NAb, neutralizing antibody; OnaA, onabotulinumtoxinA; SNR, secondary non-response.
